# Observations on the Diseases Which Appeared in the Army at St. Lucia in 1778 and 1779. To Which Are Prefixed Remarks, Calculated to Assist in Explaining the Treatment of Those Diseases. With an Appendix, Containing a Short Address to Military Gentlemen on the Means of Preserving Health in the West Indies

**Published:** 1781-11

**Authors:** 


III. *Obſervations on the diſeaſes which appeared in the army at St. Lucia in* 1778 *and* 1779. *To which are prefixed remarks, calculated to aſſiſt in explaining the treatment of thoſe diſeaſes. With an appendix, containing a ſhort addreſs to military gentlemen on the means of preſerving health in the Weſt Indies. By* John Rollo. *Small* 8vo. *Barbadoes printed; London reprinted by* C. Dilly, 1781. 160 pages. 2s.

THIS work conſiſts of two parts, each of which is ſubdivided into chapters and ſections. The author begins with a general deſcription of the iſland, to which he adds a particular account of the different places in it which may occaſionally be occupied by an army.

The iſland, we are told, repreſents a portion of ground, on which are every where placed perfect and imperfect cones of irregular heights, leaving in ſome parts a conſiderable flat, in many others others deep vallies covered with a ſtagnating water, impenetrable woods and poiſonous ſhrubs, and generally the reſort of noxious animals. The ſeaſons are much the ſame here as in the other Caribbee iſlands; but the rainy periods continue longer, and appear in different times. The particular rainy ſeaſon begins about the end of June, and continues ſeveral months. In the winter the mornings and evenings are chilly; in the ſummer months hot and ſultry. May and June, the author informs us, were exceedingly hot and diſagreeable, eſpecially when there was little wind, and in theſe months inſects of the moſt troubleſome kind over-run every place.

The winds generally blow from the N E. to the S. E. ſeldom varying to the weſtward. About the latter end of July, but more certainly in Auguſt and September, the winds blow very ſeverely, ſometimes forming a hurricane. Earthquakes are now and then perceptible, but are never of any ſeverity or duration.

The ſoil in general is rich and fertile. It produces cocoa, coffee, cotton, and ſugar cane; vegetables and fruit in plenty; cattle, poultry and fiſh are in abundance. Nor is St. Lucia deſtitute of medicinal productions; it yields ſimarouba, zinziber, caſſia fiſtularis, and the caſtor nut.

 Ipeca-

Ipecacoanha, ſquills, jalap, ſarſaparilla, and even bark, are likewiſe ſaid to be found here. The author cloſes his firſt chapter with a deſcription of a volcano in the neighbourhood of Souffrir. The ſecond chapter contains a regiſter of the weather from December 1778, to May 1779. In this regiſter no mention is made of the thermometer, or any other meteorological inſtrument, but it ſerves to ſhew the general ſtate of the atmoſphere, the direction of the winds, and the rainy days.

In the third chapter we find a liſt of diſeaſes, and an account of the number of men in health in different parts of the iſland. From this liſt it appears, that of 208 men 169 were attacked with diſeaſes, of which number 16 died. The diſeaſes were in the following proportions:

| | |
|---|---|
| Diarrhœa, - - - - - | 8 |
| Dropſy, - - - - - - | 2 |
| Dyſentery, - - - - - | 15 |
| Head-ach, - - - - - | 2 |
| Remittent fever, - - - | 63 |
| Quotidian, - - - - - | 47 |
| Tertian, - - - - - - | 32 |
| Quartan, - - - - - - | 1 |

Of the 16 who died, two were carried off by a diarrhœa, and fourteen by a remittent fever.

In

In the fourth chapter the author deſcribes the ſituations of the iſland, in which the men ſpecified in the table were fixed, and endeavours to determine which are the moſt healthy. For this purpoſe he gives a comparative view of the health of the natives compared with that of the troops. He obſerves, that at Carenage-town the people are ſhort-lived, have annual attacks of fever, yellow and meagre countenances, ſmall legs, except when œdematous, ſo that they have the appearance of perſons worn out by diſeaſe. At Gros Iſlet, we are told, the inhabitants live longer, are not ſo ſubject to diſeaſe, at leaſt not of the ſame degree or duration, and that they are fuller in the face, and more hearty. At Souffrir the inhabitants have cheerful countenances, and are nearly in a ſtate of health with thoſe of Gros Iſlet, but this, our author thinks, may be attributed to a better diet rather than ſituation. On the extenſive plain to windward of this place very few diſeaſes appear, and they are moſtly intermittents: the countenances here of the women, of the children, and even of the men, have ſome degree of reſemblance to thoſe of the European, the female has the red on her cheek, and the child has all the marks of health.

From this view of ſoldiers and inhabitants on difdifferent ſituations, Mr. Rollo concludes that the following particular parts of the iſland are the moſt favourable to Europeans. He has placed them according to their ſuperior degree of healthineſs; viz. 1. Windward of Souffrir. 2. Morne Fortune. 3. Vigie and Rock battery. 4. Situations about Gros Iſlet. In general he ſuppoſes the windward parts of the iſland, and ſuch as are not expoſed to the noxious effluvia of marſhes and woods, to be the moſt ſalutary places of abode.

In the firſt chapter of the ſecond part of the work, Mr. Rollo gives a hiſtory of the diſeaſes which prevailed amongſt the troops. He divides them into intermitting and remitting fevers, and dyſentery. Of the firſt of theſe, he obſerves that they were uſually attended with a yellowneſs of the ſkin, coſtiveneſs, and a high-coloured urine. Theſe ſymptoms were commonly more evident in quotidians than tertians. The paroxyſms were, in general, perfect, but of different durations, eſpecially in the quotidian, which was often a prelude to the remittent fever, and then the attacks became irregular and imperfect.

The remittent fever was ſometimes the conſequence of an intermittent, particularly if the latter had been neglected on its firſt attack; but in general this fever made its appearance in a different ferent manner, and in its turn it often ran into the intermittent.

Languor, proſtration of ſtrength, chillineſs, livid colour of the lips, a particular dejected look, and nauſea, were the firſt marks of this diſeaſe; and theſe were ſoon followed by anxiety, head-ach, pain of the back and loins, heat, thirſt, often a vomiting, and an increaſe of the nauſea, languor, and proſtration of ſtrength.

After the firſt remiſſion, which generally happened towards morning, all the preceding ſymptoms increaſed, with the addition of a foul tongue, a yellowneſs of the eyes, and, in ſome caſes, an univerſal tinge of the ſkin, delirium, urine in ſmall quantities and very high-coloured, imparting an offenſive ſmell, often a difficulty in voiding it, which ſometimes came to a perfect ſtoppage.

The pulſe, in the beginning of this fever, was ſeldom much affected. A remiſſion was perceptible from an abatement of the ſeverity of the ſymptoms, a gentle moiſture on the ſkin, a free diſcharge of urine, and a diminution of the yellowneſs.

An eruption about the mouth and ears with a ſwelling of the upper lip, either in this or the intermittent, happening when the fever was going off, was a certain ſign of recovery; but if it appeared when dangerous ſymptoms were preſent, our our author confidered it as greatly affifting in the unfavourable prognoftic.

Of the dyfentery, Mr. Rollo obferves, that in its attack, progrefs and terminations, it refembled that difeafe as it appeared in the army in America, except that it more remarkably affumed the form of the intermittent and remittent. A fever, we are told, often preceded the proper dyfenteric fymptoms, and was always coeval with them, though varying in its degree of feverity.

In the following chapter the author endeavours to trace the caufes of the difeafes juft now fpoken of. From certain circumftances he is inclined to fuppofe that altho' heat, cold and moifture, in certain degrees and combinations, may produce difeafes of confiderable feverity, they were not the common caufes of the difeafes he has defcribed; but that they were chiefly the confequence of marfh effluvia. It is evident, however, he obferves, that the former caufes operated fo far as to induce and facilitate the action of thefe effluvia, and to make them more univerfal and active in their effects.

It has been generally remarked, that the effluvia of marfhes are moft active when the water drains off and the earth appears. This, we are told, was certainly the cafe in St. Lucia. The greateft part part of the regular intermittents happened when the rains were moſt frequent, and before the ſtagnating pools diſcovered their bottoms; and the moſt dangerous remittents appeared when the marſhes had no water, but a ſlimy matter on their ſurface.

The third chapter contains the author's method of practice, and firſt his treatment of the intermittent.—In the abſence of the paroxyſm he exhibited a ſolution of tartar emetic, in ſmall quantities, and at ſhort intervals, to excite vomiting. After this was effected, he generally gave it in ſmaller doſes, in order to procure a few ſtools; but this depended much upon the ſtate of the patient's belly, and on the continuance of the diſeaſe. If the patient was much weakened and had a looſe belly, he omitted the emetic tartar and gave a ſmall doſe of ipecacoanha, which ſeldom produced ſtools.

He always found it neceſſary to give an emetic at the commencement of the diſeaſe, and often during its progreſs. After the operation of the emetic, he immediately gave the bark, in ſubſtance, and in as large doſes as the ſtomach could bear. This method was proſecuted even though he had only time to throw in one or two doſes of the bark before the period of the next paroxyſm; at the termination of which he began again, and in general put a ſtop to the diſeaſe in two or three paroxyſms from the firſt exhibition of the bark. If the bark was not given in this manner and in very large doſes, the fever often aſſumed a more dangerous form—that of a remittent.

The tertian was treated in the ſame manner as the quotidian, only more time was taken in clearing the ſtomach and bowels, and in attending to particular ſymptoms.—In the paroxyſm of both after the cold ſtage began to diſappear, he always gave a combination of tartar emetic and opium in ſolution; opium was given alone, if the ſtomach was very irritable; and to a careful exhibition of theſe in this ſtate of the diſeaſe, he attributes the ſucceſs he met with. The conſtant effects of this method were to abate the ſeverity of the ſymptoms, to ſhorten the duration of the paroxyſm, and to render the intermiſſion longer and more perfect. Theſe effects were more certain, if the ſtomach permitted the combination of tartar emetic.

The remittent fever required the earlieſt exhibition of medicine; for whether it appeared as a diſtinct diſeaſe immediately in its own form, or as preceded by the intermittent, it was always attended with danger; and two or three hours delay proved fatal.

If

If the remittent had been preceded by an intermittent, in which evacuations had been ufed and the difeafe was of fome continuance, Mr. Rollo feldom found it neceffary to repeat an emetic or laxative. When any of thefe evacuants were wanted, the fmalleft quantity of an antimonial or ipecacoanha was given, and common glyfters were ufed. The chief indication in fuch cafes was to procure a diftinct remiffion, or, if poffible, an intermiffion. The moft effectual means for this purpofe were found to be naufeating dofes of tartar emetic; and at the time of the ufual exacerbation of fever, an opiate by itfelf, or combined with an antimonial, according to the ftate of the ftomach, in the fame manner as after the cold ftage of an intermittent. If the febrile fymptoms ran high before the remiffion was expected, a large blifter, applied to the back, particularly if any degree of delirium was prefent, greatly affifted to bring about that defireable event.

When a remiffion was effected, our author directly adminiftered the bark. Altho' the interval allowed him perhaps to give only two or three drachms of it, or even lefs, yet in the next exacerbation the good effects of it were evident; and more fo in the fucceeding remiffion, which was longer and more perfect. In this way he continued until the fever was removed, or brought into the intermittent form. But if his attempts failed, and he had the greatest reason to dread the termination, he continued the bark, without regarding the existence of fever, and added to it the use of snake-root. If the stomach could not retain the bark, he gave a strong infusion of snake-root alone, and ordered wine, in proportion to the state of the disease. By this method, he informs us, he has often altered the dangerous appearance, and given a favourable turn to the complaint.

If the remittent fever appeared in its own form, and was not preceded by an intermittent, he gave the tartar emetic in doses sufficient to produce vomiting and purging, taking care to encourage the latter no farther than to procure an effectual discharge of the contents of the intestines, and not to weaken the patient. If the exacerbation succeeded these operations, he generally gave an opiate, and some time after reassumed the tartar emetic in nauseating doses, frequently increasing the quantity so as to produce a gentle rejection of the stomach. In cases of this sort, as the danger became sooner apparent, our author was more anxious to throw in a proper quantity of the bark; and when it was rejected by the mouth, he attempted tempted it in clyſters, but he ſeldom found his endeavours of any uſe, as the diſeaſe in this ſtate was generally beyond the power of medicine.

Towards the termination of the fever, or in the laſt ſtages of it when the patient was much reduced, he experienced the beſt effects from wine. When bark was rejected, and every bad appearance preſent, he has been ſenſible of benefit from this valuable article; but it was given from one to two pints in the courſe of twenty-four hours, in ſmall doſes, and at proper intervals. He is convinced that many, by a proper uſe of it, have eſcaped death; but at the ſame time, is aware that it has been imprudently uſed by having been given too early, or in too great a quantity.

In the treatment of the dyſentery, Mr. Rollo always began by emptying the ſtomach and bowels. For this purpoſe he generally employed a weak ſolution of emetic tartar, unleſs the patient was much reduced, or had been two or three days ill, for in ſuch caſes he generally preferred ſmall doſes of ipecacoanha, and a weak ſolution of Glauber's ſalt. After the operation of theſe medicines he gave an opiate, which in many caſes was repeated in two hours.

Next morning he preſcribed a combination of tartar emetic and opium, ſometimes in a ſolid, but but oftener in a liquid form. He has given to the quantity of three or four grains of opium in this way, in the courfe of the day, and the general confequences were large ftools; an abatement of the griping and tenefmus, and a remiffion of the febrile fymptoms. Thefe good effects were more certain, if the medicine was given fo as to excite naufea, which the author always endeavoured to maintain.

At the commencement of the treatment, he often ufed fomentations to the lower extremities and to the abdomen, which were continued till a remiffion of the pain was effected. When the griping or pain of the belly was very fevere, the application of volatile liniment and blifters was found to have a good effect.

When the dyfentery affumed the form of an intermittent, it was treated in much the fame manner as that difeafe; and when it affumed a dangerous appearance, the author's principal reliance was on the bark.

As aftringents, he tried the different preparations of earths, infufions and decoctions of fimarouba and the bark; but he found opium to be the beft, affifted by diet, air, and cleanlinefs.

After this account of the general mode of treatment, our author proceeds to treat of particular fymp-

ſymptoms. Theſe were vomiting, looſeneſs, comatoſe diſpoſition and hiccup. Of the firſt of theſe, vomiting, he obſerves that it very often occurred in the remittent fever, and ſometimes in the quotidian. Beſides preventing the exhibition of medicine, it was always accompanied with pain, and an increaſe of every attendant ſymptom; and when it took place after the diſeaſe had been of ſome continuance, it was a conſtant mark of danger.

When vomiting appeared at the commencement of the diſeaſe, it generally went off by encouraging it either with chamomile tea, or ſmall doſes of an antimonial, or a few grains of ipecacoanha. If coſtiveneſs attended, a ſtool was procured by clyſters. If this ſymptom continued, or appeared in a later period of the diſeaſe, with pain in the ſituation of the ſtomach or liver, a bliſter applied over the whole extent of the epigaſtric region, was in general attended with a very good effect. This application was aſſiſted by the uſe of ſaline draughts.

Mr. Rollo thinks that in ſome caſes he has ſeen a ſmall quantity of opium of uſe, provided the patient abſtained from liquids for ſome time after taking it. When vomiting ſucceeded the uſe of the bark, it was for awhile omitted, or given in a different form. In cafes of this kind, an opiate given in a folid ftate, about an hour or two before frefh recourfe was had to the bark, feldom failed to prevent it.

In cafes of loofenefs, if its continuance threatened danger, a free ufe of opiates combined with bark, was found to fucceed better than any other remedy that was tried.

Comatofe difpofition was generally checked in its progrefs, and a favourable turn given to the difeafe, by applying a large blifter over the whole fcalp, immediately on the appearance of the comatofe difpofition. At the fame time if the degree of danger was great, another blifter was applied to the back, or one to each ancle, while camphor was given internally in a thick folution of extract of bark every two or three hours.

In cafes of hiccup, mufk and camphor were given in large dofes, but without any good effect. When this fymptom appeared early, a gentle emetic removed it; and when it was united to other fymptoms of danger, the bark was the only medicine from which any relief was derived.

The author next fpeaks of the treatment of after-complaints, under the feveral heads of, 1. Pectoral complaints. 2. Hypochondriac affection. 3. Abdominal fwellings. 4. Œdematous fwellings fwellings of the lower extremities. 5. A weak ftate of the ftomach, with flatulence. 6. Coftivenefs. And, 7. Diarrhœa.——Thefe fecondary diforders generally took place in patients whofe difeafe had been of a long continuance. Pectoral complaints, we are told, were very frequent. The application of blifters, fmall dofes of ipecacoanha, occafionally repeated, with gentle opiates, in the form of Dover's powder, or antimony and laudanum, a milk and vegetable diet and gentle exercife, were found to be the beft remedies in fuch cafes.

In cafes of hypochondriac affection, a careful attention to prevent the patient from being by himfelf, a change of fituation, exercife, chalybeate preparations, and cold bathing, if no topical affection exifted, were the means our author found moft fuccefsful.

In abdominal fwellings, if the complaint appeared to originate from an enlargement of the liver, accompanied with pain on preffure, he began the removal of it by applying a blifter, and giving neutral falts to keep the belly gently open. When the pain went off, recourfe was had to mercurial ointment applied externally.

A weak ftate of the ftomach was generally relieved by chalybeates, mineral acids, aromatics, bitters, abforbents, abftinence from vegetables,

 exerexercife, and a free air. Coftivenefs was obviated by aloetic preparations, folutions of alkaline falt, and a laxative diet. In cafes of obftinate diarrhœa, Mr. Rollo found no medicine fo efficacious as opium. He has given it to the quantity of three or four grains a day, and is convinced of having faved many lives by it. Though he has continued the ufe of it for feveral weeks, he never met with any bad effect, or any difficulty, in difcontinuing it.

In the fourth chapter the author relates feveral cafes to illuftrate what he had advanced in the preceding chapters; after which he treats of relapfes, the caufes of which he refers to, 1. The diet of foldiers. 2. The neceffity of their returning to duty very foon after recovery. 3. The impracticability of fending them to more healthy fituations. Upon the whole he confidered a change of air, a regulated diet and exercife, and a well-conducted cold bathing, (if no topical affection exifted) as the only certain means of preventing a relapfe, by being beft calculated to reftore the natural vigour of the fyftem.

In the author's addrefs to military gentlemen, on the means of preferving health, we meet with many judicious obfervations, which cannot fail of being ufeful to thofe Europeans who vifit the Weft Indies.

